# Tuning Promoter Strength through RNA Polymerase Binding Site Design in *Escherichia coli*

**DOI:** 10.1371/journal.pcbi.1002811

**Published:** 2012-12-13

**Authors:** Robert C. Brewster, Daniel L. Jones, Rob Phillips

**Affiliations:** 1 Department of Applied Physics, California Institute of Technology, Pasadena, California, United States of America; 2 Division of Biology, California Institute of Technology, Pasadena, California, United States of America; University of Basel, Switzerland

## Abstract

One of the paramount goals of synthetic biology is to have the ability to tune transcriptional networks to targeted levels of expression at will. As a step in that direction, we have constructed a set of 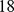 unique binding sites for *E. coli* RNA Polymerase (RNAP) 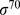 holoenzyme, designed using a model of sequence-dependent binding energy combined with a thermodynamic model of transcription to produce a targeted level of gene expression. This promoter set allows us to determine the correspondence between the absolute numbers of mRNA molecules or protein products and the predicted promoter binding energies measured in 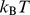 energy units. These binding sites adhere on average to the predicted level of gene expression over 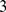 orders of magnitude in constitutive gene expression, to within a factor of 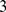 in both protein and mRNA copy number. With these promoters in hand, we then place them under the regulatory control of a bacterial repressor and show that again there is a strict correspondence between the measured and predicted levels of expression, demonstrating the transferability of the promoters to an alternate regulatory context. In particular, our thermodynamic model predicts the expression from our promoters under a range of repressor concentrations between several per cell up to over 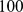 per cell. After correcting the predicted polymerase binding strength using the data from the unregulated promoter, the thermodynamic model accurately predicts the expression for the simple repression strains to within 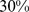. Demonstration of modular promoter design, where parts of the circuit (such as RNAP/TF binding strength and transcription factor copy number) can be independently chosen from a stock list and combined to give a predictable result, has important implications as an engineering tool for use in synthetic biology.

## Author Summary

One of the most fundamental tuning parameters governing expression of a given gene is the strength of its promoter. But what are the sequence rules that govern promoter strength? Recent high throughput mutagenesis experiments present an improved method for constructing an energy function that maps sequence to protein-DNA binding energy. We use this energy function combined with a thermodynamic model to deliberately design different promoters with over three orders of magnitude difference in their mean expression, and measure the resulting level of expression at both the mRNA and protein level to test this design strategy. The designed promoters are used in an alternate regulatory architecture and can now serve as the basis for the systematic examination of how both the mean and noise in gene expression depend upon the regulatory parameters that have been subject to evolutionary and/or human change.

## Introduction

**Figure 1:**
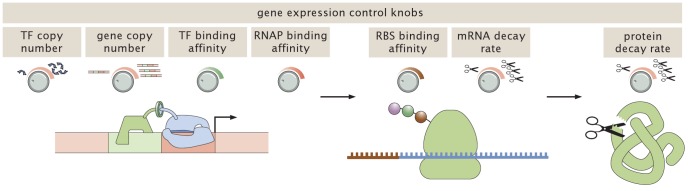
Regulatory control knobs. A schematic view of the available knobs which can be systematically tuned to change the mRNA and protein distributions. In this work we begin by studying constitutive expression, eliminating the extra layer of complexity associated with transcription factors, and systematically control the RNAP binding affinity through control of the promoter sequence. These results are then generalized to the case in which these same promoters are subjected to regulation by repressor binding, with the level of repressor (i.e. TF copy number) controlled systematically.

The regulation of gene expression is one of the primary ways that cells respond to their environments. The quantitative dissection of the networks that control such expression as well as the construction of designed networks has been a central preoccupation of regulatory biology. As sketched in Figure 1, the level of gene expression exhibited by a cell can be targeted at multiple levels along the path from DNA to protein. Key biological tuning variables include the copy number of the transcription factors that act on a gene of interest, the strength of their binding sites, the strength of RNA polymerase binding, the strength of ribosomal binding sites and the degradation rates of the protein products of the gene of interest. Many of these tuning parameters have been studied in quantitative detail. For instance, Salis *et al.* [1] developed a model to describe the interaction energy between the ribosomal binding site (RBS) of an mRNA transcript and the 30S ribosomal subunit, which they relate to translation initiation rate using statistical thermodynamics. Using this model, gene expression can be predictively tuned over 5 orders of magnitude by modulating translation efficiency for a given gene [1], [2]. Translation initiation (and hence protein expression) is thus tuned by choosing an RBS sequence with the desired interaction energy. The rate of protein degradation is another key determinant of intracellular protein concentration. Protein degradation can be modulated by the use of degradation tags appended to the C-terminal domain of a given protein. The ssrA tag [3], for instance, targets proteins for destruction by the *E. coli* degradation machinery, which includes proteases ClpXP, ClpAP and SspB [4]. This degradation system has been artificially implemented in yeast, where ClpXP is expressed from an inducible promoter, and degradation rates of ssrA-tagged proteins can be tuned over a factor of 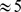 by controlling the ClpXP concentration in the cell [5]. Similarly, manipulating the decay rate of the protein's transcript allows for modulation of the steady-state protein copy number [6], [7].

In this paper, we focus on two sets of these transcriptional parameters: namely, the strength with which polymerase binds the promoter, and the number of transcription factors present when that promoter is controlled by simple repression. We begin by focusing on the simplest case where there are no repressor proteins present in the cell. Our interest in such “constitutive” promoters (those not regulated by transcription factors) stems from the goal of creating a set of promoters in which we can systematically vary both the mean and the noise to test recent models of transcriptional kinetics [8]. These experiments are further motivated by measurements which question our understanding of how the mean and noise in transcription depend on the architecture of the promoter [9]. To test these ideas on noise in transcription, we must know how to predictively tune the binding strength of RNAP to the promoter.

**Figure 2:**
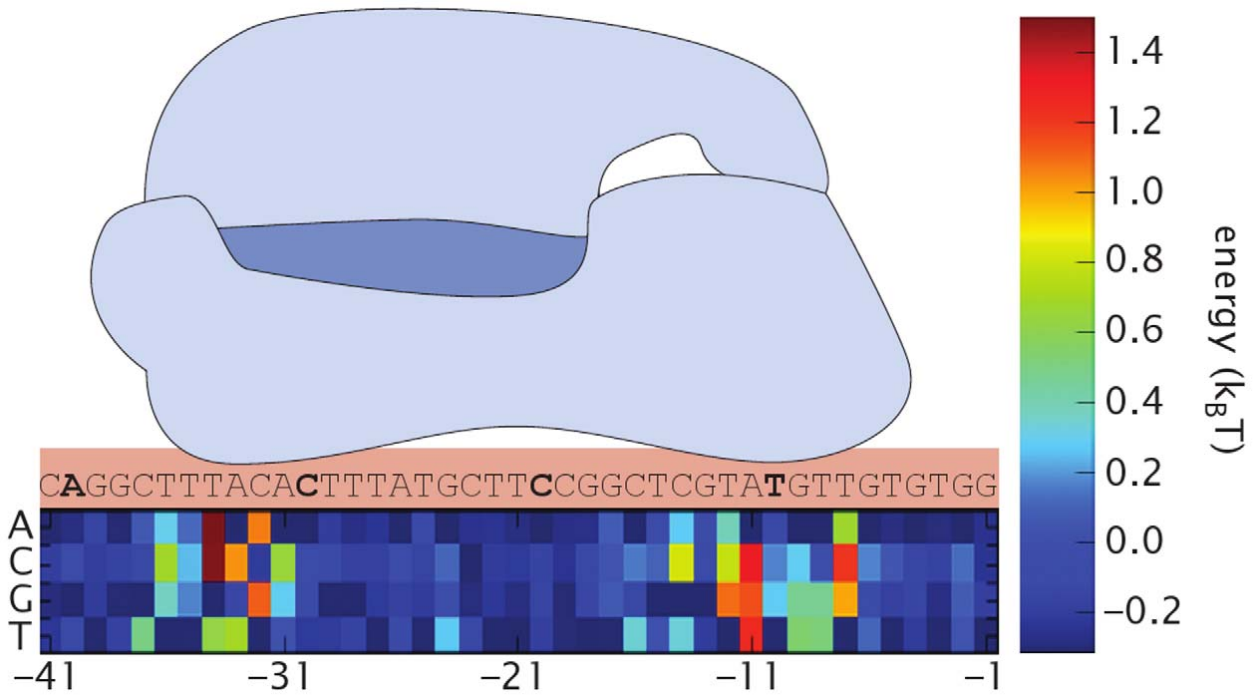
Energy matrix for RNAP binding. Figure adapted from Kinney *et al* [20]. The contribution of each basepair to the total binding energy is represented by color. The total binding energy of a particular sequence can be calculated by summing the contribution from each base pair. Positive values indicate disfavorable contributions to binding energy. As expected, the most influential base pairs are those in the 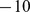 and 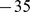 region which interact directly with the binding domains of RNAP 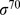. Numeric matrix entries are available in SI Text S2. The sequence displayed above the energy matrix corresponds to the wild-type *lac* promoter; the bold bases mark 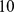 base pair increments. 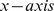 coordinates are with respect to the transcription start site.

Precise physical modelling of protein-DNA interaction energies is a difficult problem involving many degrees of freedom. Such binding energies are at the heart of the molecular interactions which result in (or, in the case of repressor transcription factors, prevent) transcription events. Hence, precise control of protein-DNA binding is an essential prerequisite for quantitative control of transcription. Despite the complexity of protein-DNA interactions and numerous molecular mechanisms involved in transcription initiation [10]–[14], simple linear models of sequence-dependent binding energies are often sufficient to describe the interactions of transcription factors (TFs) or RNAP with DNA [15]–[20]. A “linear model” treats each base along the binding site as independently contributing a defined amount to the total binding energy. The total binding energy is then the sum of the contributions from each base along the binding site. In one recent study, the authors inferred the 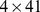 parameters describing the interaction of RNAP 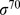 holoenzyme with DNA [20]. This matrix is shown pictorially in Figure 2 and the numerical values are provided in Supporting Information (SI) Text S2. Mathematically, the binding energy of RNAP to a specific sequence is calculated using a matrix 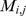 of 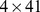 energy values where 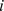 represents the base identity (A,C,T,G), and 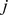 represents the base pair position along the binding site. For instance, 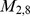 represents the contribution from having a “C” present at position 8 along the binding site. We represent a particular promoter sequence by a 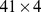 matrix 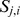 which is unity if the 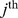 base pair has identity 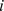 and zero otherwise. The total energy of the sequence in question is the inner product of these matrices, namely,
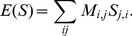
(1)For convenience, we have added a constant offset to the matrix such that the average value of 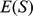 across the *E. coli* genome is zero (see SI Text S1 for the original matrix from ref. [20], SI Text S2 for the adapted matrix, and SI Text S3 for the Python source code to perform the adaptation). Since only differences in energy (such as between two different promoter sequences) are physically meaningful, we can add the same constant value to each element of the matrix without affecting its physical interpretation.

We use this correspondence between promoter sequence and RNAP binding affinity to generate a suite of promoters with a wide range of binding affinities. We then show how a simple thermodynamic model of transcription, which postulates that transcriptional activity is proportional to the probability of finding the RNAP bound at the promoter, accurately predicts the scaling of the expression with RNAP binding energy. In addition, these measurements allow us to determine the proportionality between RNAP binding probability and transcriptional output for our gene. With this information, we can make absolute predictions for the transcriptional output of our designed promoters under other regulatory conditions. We test and confirm these predictions by measuring the transcriptional output of some of our promoters in the architectural context of simple repression (similar to Ref. [2]) and show we are able to make accurate, absolute predictions of the transcription as a function of average repressor copy number.

## Results

**Figure 3:**
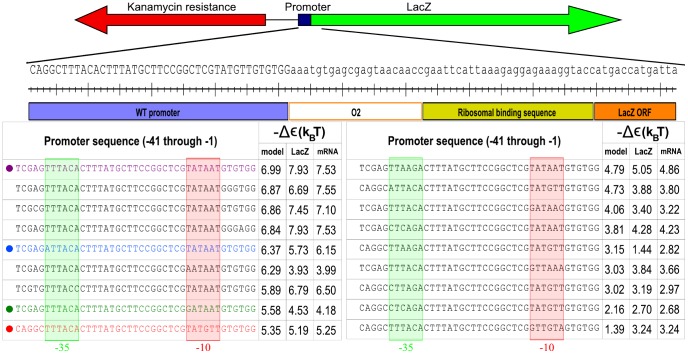
Schematic of DNA construct inserted in the *galK* region. The area between the promoter and the LacZ start codon is shown in more detail below along with a table displaying the specific RNAP binding sites (promoters) listed in order of descending binding affinity. The wild-type binding sequence is shown in red text, the *lac*UV5 sequence is shown in magenta text, and two additional promoters are marked by blue text and green text. The data points involving these four promoters will maintain this color coding throughout every figure. The 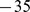 and 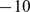 RNAP recognition sequences are highlighted in a green box and a red box, respectively. The bases in these regions carry the most weight in the energy matrix. Sequences are available in text format in SI Text S4.

We set out to design sets of unique RNAP sites with specific binding energies separated by 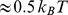 steps. Taking as a starting point the wild-type *lac* and *lac*UV5 promoters, we used the RNAP binding energy model in Figure 2 to choose appropriate base pair mutations (concentrated in the −10 and −35 boxes, where mutations carry the most weight) which result in our desired energy levels. The 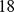 strains designed by this process have binding energies spanning roughly 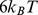 and levels of constitutive gene expression from roughly 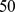 times less to 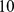 times greater than that of the wild-type *lac* operon. The specific sequences of these 18 promoters are listed in the table shown in Figure 3 along with their predicted “model” RNAP binding energy for that sequence. Four promoters are marked with a colored dot; this color coding will be preserved throughout every figure. While the *lac*O2 site is present in our reporter construct, the strain used to measure constitutive expression does not produce LacI, the repressor which specifically binds to this site (see methods). In addition, the CRP binding site which would otherwise serve to activate the *lac* promoter has been removed. Based on intuition from thermodynamic models of transcription regulation [21]–[25], we expect that the expression level of a given promoter will scale with the probability that RNAP is bound at that promoter. A derivation of this probability as a function of RNAP binding energy for our promoter architecture is shown below. To test the predictive power of our design process in conjunction with the thermodynamic model, we used single-cell mRNA fluorescence in-situ hybridization (mRNA FISH) and a colorimetric enzymatic assay to measure, for each construct, the average mRNA and protein copy number per cell of LacZ reporter. We then compared these results with those predicted by the calculated RNAP binding energy of that promoter. Finally, we use this same strategy to examine simple repression in the context of our designed promoters.

### Thermodynamic Model for Constitutive Expression

**Figure 4:**
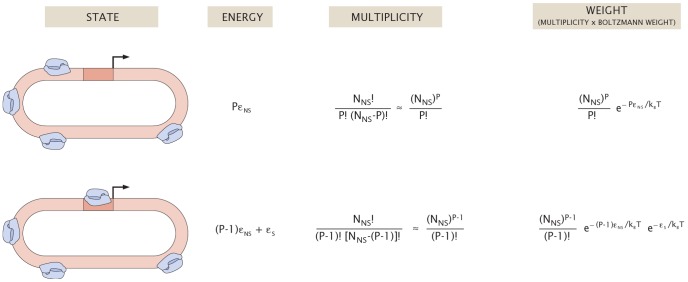
States and weights of the unregulated promoter. In the thermodynamic model, the promoter can be in one of two configurations: unoccupied by RNA polymerase (top) or occupied by RNA polymerase (bottom). The remaining polymerases are bound nonspecifically on the *E. coli* genome. The total energy is the sum of all the nonspecific binding energies and the specific energy of binding at the promoter (when occupied). The multiplicity factor accounts for the number of different ways of arranging polymerases on the genome.

To construct promoters with a targeted level of gene expression, we compute the RNAP binding probability using a simple thermodynamic model based upon the RNAP binding energy matrix from the work of Kinney *et al* [20] (shown in Figure 2). A schematic of the allowed microscopic states of the promoter in the constitutive expression system, along with their thermodynamic weights, is shown in Figure 4. This model treats all non-specific binding sites (i.e., binding sites other than the promoter of interest) as binding RNAP with a fixed energy 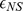. More nuanced treatments of the non-specific background can be found in Refs. [19], [26], [27], for example. Consider a cell with 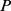 RNAP molecules which can bind non-specifically with energy 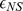 to 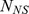 non-specific RNAP binding sites and with energy 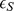 to the promoter of interest [21]–[25]. The energy of the state in which the promoter is unoccupied is 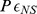 which can occur in 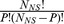 unique configurations. Similarly, the energy of the state in which RNAP is specifically bound is given by 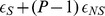, and its multiplicity is given by 
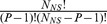
. The probability that RNAP is bound is the Boltzmann factor of the bound state normalized by the partition function of the system, which simplifies to
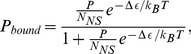
(2)where 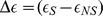 and where we have used the fact that 
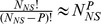
 for 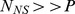. In the simplifying case of a “weak promoter”, where 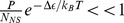, this expression reduces to
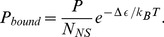
(3)Note that the microscopic language used to make these derivations is convenient for interpreting binding energies and the dependence on number of polymerases. However, all of these results can be naturally derived and written in the alternative language of dissociation constants without ever making reference to the nonspecific background [23]. For example, we can write
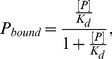
(4)where 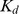 is the *in vivo* dissociation constant for RNAP from the promoter of interest.

With these results, we can now explore the connection between the measured and the corresponding predicted level of expression. Since gene expression is (by assumption) proportional to 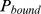, we can use equation 3 to conclude that
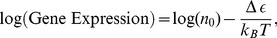
(5)where 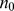 is an unknown constant of proportionality related to the number of mRNA or proteins expected from a promoter with 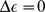. With this relation in hand, we are now equipped to take the predicted energy for each RNAP binding site and compare the resulting expression to that predicted from equation 5.

### Constitutive Gene Expression Measurements: mRNA and Protein

**Figure 5:**
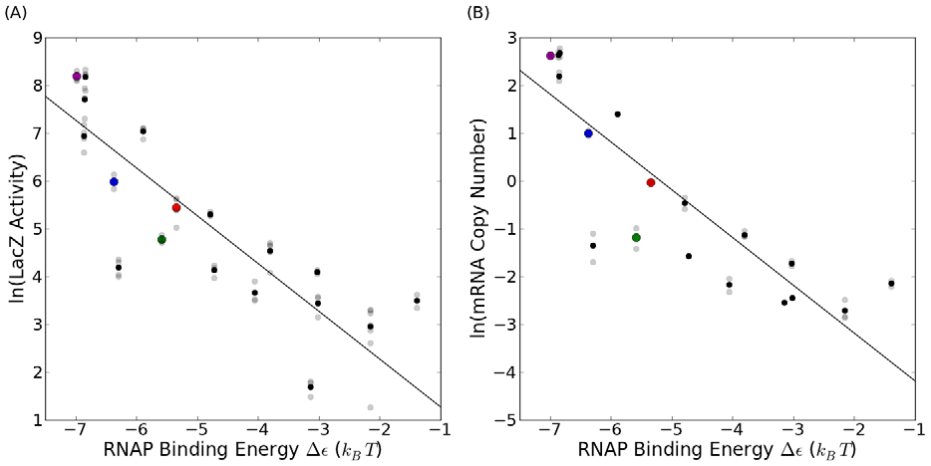
Gene expression as a function of RNAP binding energy. (A) LacZ activity measured in Miller units and (B) average mRNA per cell vs. promoter binding energy in units of 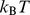 (with the zero of energy set to be the average interaction energy between RNAP and the the entire *E. coli* chromosome). To illustrate the reproducibility of our measurements, the translucent points represent individual measurements and the solid points represent the averaged value over repeated experiments. The solid black line in each plot is the Boltzmann factor scaling, 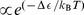. The red data points correspond to the wild-type *lac* promoter, which was used to calibrate the arbitrary units of our energy matrix to (physical) 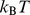 units. The magenta, red, blue, and green data points represent promoters which we examine in the context of simple repression.

To test the predictive power of the binding energy model, we measured protein expression and mRNA copy numbers for constitutive expression from each of our unique promoters. Based on equation 5, a semi-log plot of these data against their respective predicted binding energies in units of 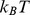 should fall along a straight line with slope equal to −1, consistent with Boltzmann scaling. Indeed, with the unknown constant 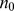 as our single fit parameter, we find that gene expression follows the exponential relation predicted from the thermodynamic model in equation 5, as seen in Figure 5. In this figure, we have taken the zero of energy to be the average energy of RNAP binding across the whole *E. coli* genome calculated from the energy matrix of Figure 2, as detailed in the Methods section below. The root-mean-square deviations of our fits are 1.02 for mRNA and 1.06 for protein. Since these values are the deviations of the natural logarithm of gene expression, we must exponentiate them to get a sense of the deviation in physical units. We conclude that our design process accurately predicts expression to within a factor of 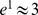 over nearly three orders of magnitude. In addition, the table in Figure 3 shows the predicted energy for each promoter (the column labelled “Model”), calculated using the matrix in Figure 2, as well as the experimentally measured energies of each promoter. To compute these measured energies, we solve equation 5 for 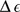, yielding 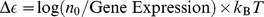. We then plug in the measured expression for each promoter and the inferred value for 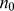 (the 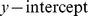 of the black line in Figure 5) to compute 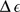 for each promoter. The measured values for the RNAP binding energies for the LacZ and mRNA data are listed in Figure 3. The promoters with colored entries will be further examined in the context of simple repression later in this work. The direct correlation between these two measurements of gene expression are shown in SI Figure S1 where protein expression is plotted vs average mRNA copy number for every promoter strength, exhibiting an excellent linear relation between these two readouts of expression.

Fitting the data in Figure 5 to the full form for 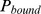 in equation 2, allowing both 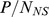 and the unknown proportionality constant between 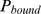 to vary, we find 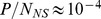 for both the mRNA and the protein data. This is consistent with typical values for RNA polymerase copy number and the length of the *E. coli* genome (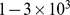 [28]–[31] and 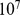, respectively), and thus the weak promoter limit appears to hold over the range of promoter strengths tested.

### Protein Burst Size

Since mRNA and protein are linked by translation, their levels for a given promoter should be related. Individual mRNAs can be translated multiple times and it has been shown that the number of translations per mRNA is well described by an exponential distribution with mean 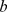, known as the protein burst size, which is the average number of proteins produced per mRNA [8], [32], [33]. Using the data described above, we can extract the burst size, defined as the ratio of protein production rate and the mRNA production rate, 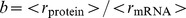 [8], [34]. The quantity we measure, however, is the steady-state copy number 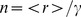, where 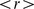 is the average rate of mRNA or protein production and 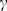 is the associated decay rate. Figures 5A and B demonstrate that the copy number 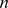 is well described by Boltzmann scaling with 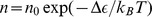. Using this knowledge, we rewrite the burst size as
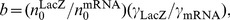
(6)with 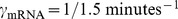 [35] and 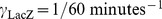 (equal to the inverse of the cell division time). This gives us a measurement of the LacZ activity (measured in Miller units, described in the methods section) per mRNA; from available biochemical data we convert from Miller units to number of LacZ tetramers [36]–[39] (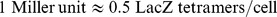 [39]). Plugging these values into equation 6 we find the protein burst size, 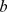, for the particular RBS we have used is roughly 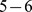 LacZ tetramers or 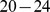 individual LacZ proteins per mRNA.

### Thermodynamic Model for Simple Repression

Our discussion so far has focused on the behavior of the designed promoters in the absence of any regulatory interventions. We were interested in examining the portability of these promoters to other contexts such as when they are regulated by transcription factor binding. In the *E. coli* genome, there are hundreds of genes that are regulated by motifs involving simple repression [40]. For these architectures, there is a single binding site for a repressor protein which reduces the expression from the gene of interest.

**Figure 6:**
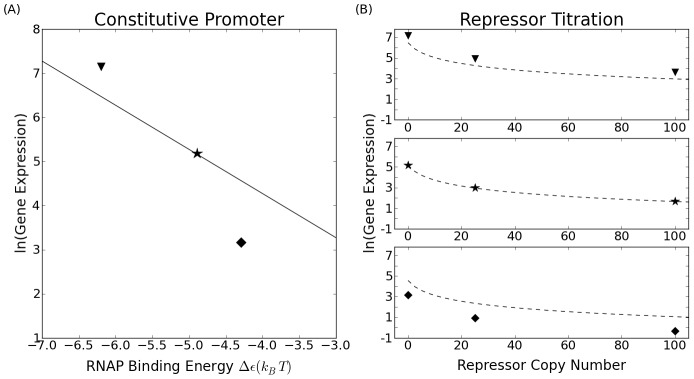
Expected relation between predictions and measurement for simple repressor titration. Figure (A) shows three hypothetical promoters for which the predictions of the promoter design are either numerically correct (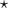), underestimated (▾) or overestimated (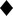). The three smaller figures in (B) show the expected result as repressors are added in a simple repression architecture. The predicted theory line and the data points differ on average by the same percent as they do at 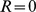.

Addition of a repressor which binds to a proximal binding site necessitates the addition of a term to the partition function of the RNAP binding probability given by equation 2. This additional term corresponds to the probability of repressor binding and making the promoter unavailable to polymerase. The resulting expression level in the context of thermodynamic models is then given by
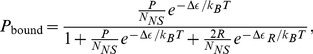
(7)where 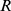 is the number of repressors (the factor of 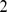 originates from the fact that LacI has two binding heads) and 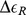 is the binding strength of that repressor to the specific binding site [2], [25]. In the weak promoter limit the expression can be simplified to,
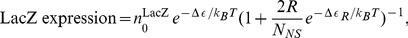
(8)where, 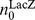, was determined in the previous section by fitting equation 5 to the constitutive expression data in Figure 5A. We therefore have an absolute prediction for the level of gene expression in our LacZ measurements. The prefactor 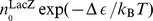 is the constitutive (R = 0) prediction for expression. It is a constant prefactor for all values of R (at a given promoter strength) and thus the model predicts that any discrepancies between predicted and measured RNAP binding energies will be inherited through all repressor concentrations. This point is illustrated in Figure 6 where we show how the repressor titration predictions depend upon how well the original constitutive promoters follow the simple Boltzmann scaling. In particular, we show the level of expression for three hypothetical promoters, one whose constitutive properties are underestimated, one whose constitutive properties are overestimated and one for which the Boltzmann scaling is obeyed precisely. What we see is that the repressor titration (Figure 6B) inherits the error already present in the constitutive promoters from incorrectly predicting the RNAP binding energy.

### Gene Expression in Simple Repression

**Figure 7:**
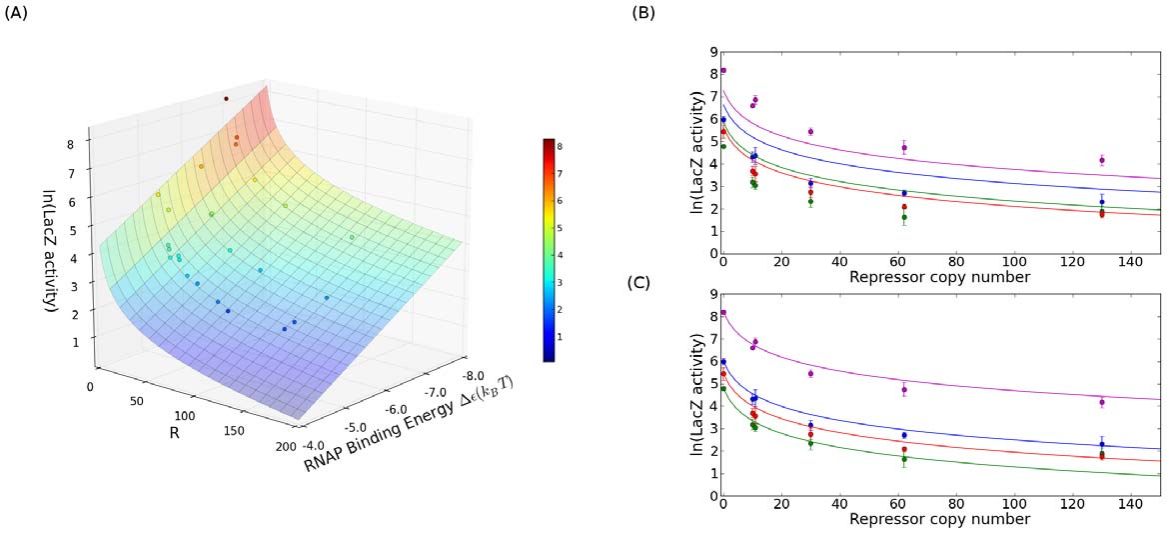
Gene expression in the simple repression case. (A) Solid surface: predicted gene expression of equation 7 as a function of repressor copy number 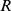 and RNAP binding energy 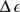. Data points represent measurements of gene expression in a strain with a given promoter and repressor copy number. (B) Data from part (A) collapsed onto the RNAP binding energy axis. The solid lines are the zero parameter predictions from the theory in equation 7 using 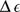 predicted from the position-weight matrix in Figure 2 (numerical values listed in Figure 3 under “model”). There is a systematic deviation between the theory and the experimental data which is inherited from the imperfect prediction of 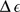 by the RNAP binding strength model (illustrated schematically in Figure 6. In (c) the same data are shown after we have corrected 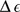 to fall on the theory fit line based on the constitutive expression (numerical values listed in Figure 3 under “LacZ”). Here we see that by correcting for the initial uncertainty in the binding energy prediction we observe good agreement between the theory and experimental data which indicates that our designed promoters function as expected even in a different regulatory context.

In each of our strains, the LacI O2 binding site is present near the promoter (see Figure 3). We reintroduce the repressor into our strains by integrating a cassette into the genome which expresses LacI. Specific LacI concentrations are obtained through modulation of the ribosomal binding sequence of the LacI gene. Using this process we create 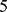 unique strains with average LacI copy numbers between 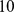 and 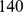 repressors per cell. Using equation 8, we can make parameter-free predictions for the overall level of gene expression as a function of promoter strength, repressor binding strength and repressor copy number for the simple repression architecture. In Figure 7A, we show a comparison between predicted and measured protein expression in the case of simple repression, as a function of repressor copy number and of predicted promoter binding strength (using 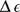 from the “model” column of Figure 3, and 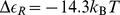 as found in Ref. [2]). Our measurements (using the same LacZ assay as for the constitutive data above) for three distinct promoters along with data from the *lac*UV5 promoter (from Ref. [2]) are shown as points color coded by expression level; Figure 7B shows the same comparison between theory and experiment collapsed along the promoter-strength axis. Each color represents a different promoter strength, with points representing measurements and the solid line representing the theoretical prediction for that promoter.

The data in Figure 7B show a clear trend, for any one promoter, to either over or under predict the expression as was sketched in Figure 6. We attribute this to imperfect predictive powers of the RNAP binding energy model from Kinney *et al* (shown in Figure 2) [20]: if the thermodynamic theory underpredicts the measured expression at R = 0 using the model value for the RNAP binding energy (for instance, the magenta point in Figure 5A), the theory will continue to underpredict the measured expression as repressors are added (as seen for the magenta points in Figure 7B). In Figure 7(C) we show the result of using the measured RNAP binding energies (from the column labelled “LacZ” in Figure 3) for the promoter binding strength and the accordance between theory and experimental data is evident. It is clear from these measurements that our promoter library exhibits the kind of “transferability” required in order to use them in different regulatory contexts. In particular, the comparison between theory and experiment is very favorable even for the repressed architectures and the imperfect agreement is actually primarily an inheritance of the imperfect accord between theory and experiment for the unregulated promoters themselves.

## Discussion

In this paper, we have shown how high throughput data obtained from experiments like those in Ref. [20] provide a foundation that, together with quantitative predictions from simple thermodynamic models [21]–[25], can be used to *predictively* tune protein-DNA interactions to produce a desired output from a gene with high precision. This approach contrasts with previous promoter engineering efforts, which have typically relied upon generating promoter libraries using random mutagenesis, followed by selection for mutants with desired expression levels [41]–[43]. We believe that predictive, model-based engineering of promoters represents a significant technical improvement over random mutagenesis, and moreover points the way to simultaneously engineering multiple aspects of promoter function (such as repressor or activator binding strengths) in a scalable way. We demonstrate the validity of our approach by simultaneously varying RNAP-promoter binding strength and the copy number of a transcription factor that represses these promoters. In this case, we can predict the absolute level of gene expression (once the conversion constant between binding probability and expression units, 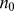, is known) as a function of transcription factor concentration.

While the binding site design procedure described here focused on alterations to the −10 and −35 region of promoters, we have made preliminary studies in which promoters are subjected to more severe perturbations, which indicate that the energy function does not describe these situations nearly so well. It is clear that changes in the linker region can have subtle effects on the twist registry and absolute spacing of the −10 and −35 binding sites that are not well accounted for by a linear weight matrix, which ignores correlations in multiple basepair changes [44]. Despite these challenges, constitutive expression from promoters designed in this study agrees well with the scaling predicted from the simple thermodynamic model presented here, and we have shown that our knowledge of simple repression can be applied on top of our understanding of constitutive expression to accurately predict the absolute expression from a gene when repression is introduced.

## Methods

### Energy Matrix

The energy matrix from [20] is given in arbitrary energy units (AU). To calibrate these arbitrary units to physical units, we need two known reference energies, since only differences in energy are physically significant. From [45], we know that RNAP binds the wild-type (WT) *lac* promoter with a binding energy 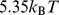 more favorable than the non-specific background. Using the matrix from [20], we find that the wild-type *lac* promoter has a binding energy of 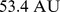, while the average binding energy of all 41 bp segments in the *E. coli* strain MG1655 is 91.3 AU (recall that the more positive the energy value, the less favorable the binding interaction). To obtain this value, we began at the chromosomal origin of replication and applied the matrix sequentially to each 41 bp segment (both forward and reverse strands) around the chromosome, and computed the mean of the resulting 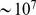 energy values. Thus, we find that a difference of 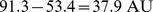 is equivalent to a difference of 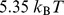, providing us with a conversion factor of 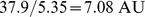 per 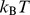.

To see how this plays out in practice, consider a hypothetical sequence whose binding energy is computed to be 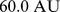. The number we are actually interested in is 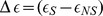. For this promoter sequence, we find that 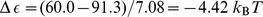. We used the same approach to convert from AU to the 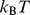 units on the 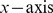 of Figure 5 for each of our distinct promoter sequences.

### Strains

All strains used are wild-type *E.coli* (MG1655) with a complete deletion of the *lacIZYA* genes [39]. Modified promoters are created through site-directed mutagenesis of plasmid pZS2502+11-lacz [2], [46], which has the *lac*UV5 promoter expressing LacZ (our reporter gene). These constructs are then integrated into the *galK* region using recombineering [47]. A schematic of the integrated region is shown in Figure 3. The end result is a strain with a desired, multi-basepair change to the *lac*UV5 promoter which expresses LacZ and a complete deletion of the LacI protein. Our designed promoters span roughly 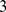 orders of magnitude in constitutive expression and vary from the wild-type promoter by as few as 1 or as many as 9 individual basepair changes. The site labelled “O2” is a binding site for the LacI repressor protein.

For the strains involving simple repression, we took our constitutive expression strains and created as many as 8 different strains with the LacI cassettes from Ref. [2] integrated at the *ybcN* site. The cassettes contain LacI expressed from an unregulated *tet* promoter with unique ribosomal binding sequences to produce varying LacI copy numbers. The exception is the data point at average LacI copy number of 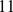, which corresponds to the native wild-type LacI gene. The measurements for repressors per cell are from quantitative immunoblots in Ref [2]. One of our strains, the one with 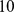 repressors/cells, has not been characterized this way, but instead the repressors/cell has been inferred from the measured expression of the *lac*UV5 promoter.

### Growth

Cultures were grown overnight (at least 8 hours) in LB and diluted 1∶4000 into 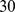 mL of M9 minimal media supplemented with 0.5% glucose in a 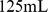 baffled flask. Cells were grown approximately 8 hours and harvested in exponential phase when OD600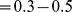 was reached.

### LacZ Assay

Our assay for measuring LacZ activity is the same as described in Ref. [2], which is a slightly modified version of that described in Ref [36]. A volume of cells from each sample between 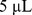 and 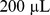 was added to Z-buffer (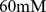 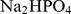, 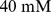 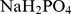, 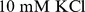, 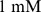 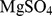, 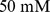 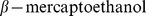, pH 7.0) to reach a total of 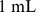. This volume is chosen to minimize the uncertainty in measuring the time of reaction (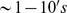 of hours) and the yellow color is easily distinguishable from a blank sample of 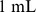 of Z-buffer. The assay was performed in 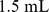 Eppendorf tubes. The cells were lysed by addition of 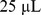 of 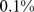 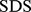 followed by 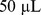 of chloroform, mixed by a 10 s vortex. The reaction was started with the addition of 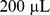 of 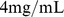 2-nitrophenyl 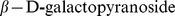 (ONPG) in Z-buffer. The developing yellow color (proportional to the concentration of the product ONP) was monitored visually. Once sufficient yellow had developed in a tube (easily measurable by OD550 and OD420, without saturating the reading), the reaction was stopped by adding 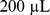 of 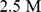 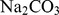. (Typically 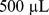 of a 1 M solution is added in other protocols, but this change allows for the entire reaction to take place in a 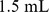 Eppendorf tube.) Once all samples were stopped, the tubes were spun at 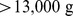 for 3 min in order to reduce the contribution of cell debris to the measurement. 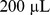 of each sample were loaded into a 96 well plate and OD420 and OD550 measurements were taken on a Tecan Safire2 with the Z-buffer sample as a blank. In addition, the OD600 of 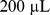 of each culture was taken with the same instrument. The absolute activity of LacZ is measured in Miller units,
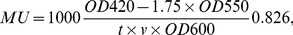
(9)where 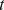 is the reaction time in minutes, 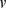 is the volume of cells used in milliliters and OD refers to the optical density measurements obtained from the plate reader. The factor of 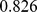 accounts for the use of 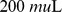 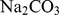 as opposed to 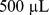 which changes the concentration of ONP in the final solution.

### Single Cell mRNA FISH

Our assay is based on that used in Ref. [9]. Once a culture reaches OD600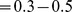, it is immersed in ice for 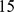 minutes before being harvested in a large centrifuge chilled to 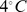 for 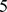 minutes at 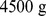. The cells are then fixed by resuspending in 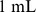 of 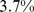 formaldehyde in 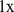 PBS which is then allowed to mix gently at room temperature for 30 minutes. Next, they are centrifuged (8 minutes at 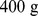) and washed twice in 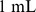 of 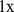 PBS twice. The cells are permeabilized by resuspension in 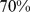 Ethanol which proceeds, with mixing, for 1 hour at room temperature. The cells are then pelleted (centrifuge at 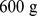 for 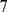 minutes) and resuspended in 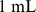 of 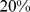 wash solution (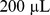 formamide, 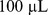 20× SSC, 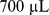 water) and resuspended in 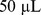 of DNA probes (consisting of an mix of 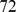 unique DNA probes, individual oligo sequences available as SI Text S5) labelled with ATTO532 dye (Atto-tec) in hybridization solution (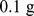 dextran sulfate, 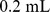 formamide, 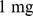 *E.coli* tRNA, 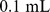 20× SSC, 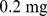 BSA, 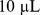 of 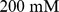 Ribonucleoside vanadyl complex). This hybridization reaction is allowed to proceed overnight. The hybridized product is then washed four times in 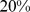 wash solution before imaging in 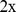 SSC.

### FISH Data Acquisition

Samples are imaged on a 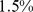 agarose pad made from PBS buffer. Each field of view is imaged with phase contrast at the focal plane and with 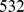 nm epifluorescence (Verdi V2 laser, Coherent Inc.) both at the focal plane and in 8 z-slices spaced 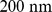 above and below the focal plane, sufficient to cover the entire depth of the *E. coli*. The images are taken with an EMCCD camera (Andor Ixon2). The phase image is used for cell segmentation and the fluorescence images are used in mRNA detection. A total of 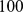 unique fields of view are imaged in each sample and a typical field of view has between 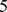 and 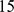 viable cells (cells which are touching and cells that have visibly begun to divide are ignored) resulting in roughly 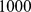 individual cells per sample.

### FISH Analysis

The FISH data is analyzed in a series of Matlab (The Mathworks) routines. The overview of the workflow is as follows: identifying individual cells, segmenting the fluorescence to identify possible mRNA, quantifying the mRNA which are found (because of the small size of *E. coli*, at high copy number mRNA can be difficult to distinguish and count by eye).

#### Cell identification and segmentation

In phase contrast imaging, *E. coli* are easily distinguishable from the background and automated programs can identify, segment and label cells with high fidelity. The results of the phase segmentation are manually checked for accuracy and bad segmentations are rejected. Cells which are touching or overlapping other cells, misidentification of cells or their boundaries or cells which have visibly begun to undergo division, etc are all discarded manually.

#### Fluorescence segmentation

First we perform several steps to process the raw intensity images. The images are flattened, a process to correct for any uneven elements in the illumination profile, using a fluorescence image of an agarose pad coated with a small drop of fluorescein (such that the drop spreads evenly across most of the pad), each pixel of every fluorescence image is scaled such that the corresponding pixel in the flattening image would be a uniform brightness (typically each pixel is scaled up to the level of the brightest pixel). This can be achieved by renormalizing each pixel in the data images and dividing by the ratio of the intensity of the corresponding pixel in the flattening image to the intensity of the brightest pixel. For instance, if one pixel in the flattening image was half as bright as the brightest pixel, the signal at that pixel's position in the raw intensity images would be doubled. We then subtract from every pixel the contribution to our signal associated with autofluorescence. The value for the autofluorescence is obtained by averaging over the fluorescence of every pixel in a control sample (one which underwent the entire FISH protocol but did not possess the *LacZ* gene). Finally, all local 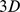 maxima (where 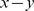 is the image plane) in fluorescence are identified. We require that the maxima be above a threshold in fluorescence (typically 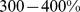 above the background autofluorescence signal). This threshold eliminates all fluorescence maxima in the control sample, which does not contain the *LacZ* gene.

#### mRNA quantification

Each identified maximum pixel is dilated in the image plane to a 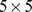 box of surrounding pixels. If this causes maxima (herein called “spots” to avoid confusion) to overlap, the pixels which make up each overlapping spot are merged into one larger spot to avoid double counting the signal from any one pixel. Since, due to the small size of the *E. coli* we can not guarantee that every spot corresponds to exactly one mRNA, we must divide the total summed intensity of each spot by the average intensity produced from a single mRNA. This value can be found by taking the average of the unmerged spots in very low expression samples (where the mean 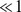 and mRNA are statistically very unlikely to overlap). We use several of our low expression strains to ensure that as we increase the mean expression it simply increases the frequency of spots with the single mRNA intensity but does not increase the mean intensity of each spot. The mean mRNA copy number can then be calculated by dividing each spot by the single mRNA intensity and averaging the total number of such mRNA in the entire collection of cells for each sample.

## Supporting Information

Figure S1
**mRNA vs. protein expression.** Scatter plot of mRNA vs. protein expression for each of our designed promoters. Each data point represents mRNA and protein expression measurements for a particular promoter. To obtain these values, expression of a LacZ reporter was measured at both the mRNA level (using mRNA FISH) and protein level (using the Miller assay of LacZ activity described in the methods). As would be expected from a simple model in which each mRNA produces a “burst” of translated protein molecules characterized by a fixed “burst size” 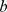, these dual measurements display a linear relationship. The inset pictures are representative mRNA FISH images from the indicated strains. The scale bar is 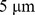.(EPS)

Text S1
**Energy matrix for RNAP** 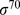 **binding affinity.** Energy matrix for RNAP 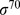 in arbitrary energy units. The energy matrix is determined from experiments in strain TK310 with no supplemental cAMP which means that these cells have no CRP. The matrix covers base pairs [−41:−1] where 0 denotes the transcription start site. Each row corresponds to a given position; each column corresponds to a value for that base pair. The columns are ordered [A,C,G,T].(TXT)

Text S2
**Energy matrix for RNAP** 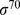 **binding affinity.** Energy matrix for RNAP 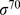 in units of 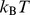. The numerical values here are shown pictorially in Figure 2. The matrix covers base pairs [−41:−1] where 0 denotes the transcription start site. Each row corresponds to a given position; each column corresponds to a value for that base pair. The columns are ordered [A,C,G,T].(TXT)

Text S3
**Source code to adapt energy matrix from Kinney** ***et. al*** **** [**20**] **.** This code converts from the arbitrary units of SI text S1 to the values in units of 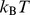 as in SI text S2. This code adds a constant offset to the matrix such that the average value of 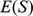 across the *E. coli* genome is zero. The basis for this conversion is the reference of 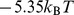 [45] for the binding energy of the wild-type promoter.(TXT)

Text S4
**Promoter sequence for constitutive expression strains.** This spreadsheet contains the colloquial name and promoter sequence for each of the unique constitutive expression strains generated for this study. The following column contains the calculated energy for each promoter using the energy matrix in SI text S1 (from [20]). The final column is the result for the binding affinity of each promoter in units of 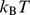 and zeroed to the *E. coli* chromosome using the energy matrix given in Figure 2 and SI text S2, as described in the methods section.(TXT)

Text S5 **List of FISH probe sequences.** A list of all 72 probes and their sequences used in the mRNA FISH protocol.
(TXT)
